# CALCULATION OF TOTAL ENERGY EXPENDITURE IN ADULTS WITH CROHN’S DISEASE BY INDIRECT CALORIMETRY AND SIMPLE WEIGHT-BASED EQUATIONS: A COMPARATIVE STUDY

**DOI:** 10.1590/S0004-2803.24612024-122

**Published:** 2025-06-16

**Authors:** Giovanna Paula de MENEZES, Cristina Eliza de Macena SOBREIRA, Maria Carolina Gonçalves DIAS, Carolina Bortolozzo Graciolli FACANALI, André Dong Won LEE, Carlos Walter SOBRADO

**Affiliations:** 1 Hospital das Clínicas da Faculdade de Medicina da Universidade de São Paulo, Divisão de Nutrição e Dietética, São Paulo, SP, Brasil.; 2 Hospital das Clínicas da Faculdade de Medicina da Universidade de São Paulo, Departamento de Cirurgia Digestiva e Gastroenterologia, São Paulo, SP, Brasil.

**Keywords:** Crohn’s disease, indirect calorimetry, energy metabolism, Doença de Crohn, calorimetria indireta, metabolismo energético

## Abstract

**Background:**

Patients diagnosed with Crohn’s disease (CD) are at high risk of nutritional impairment due to the symptoms and the intense inflammatory response of the disease. The use of indirect calorimetry (IC) to assess resting energy expenditure (REE) proves to be a valuable alternative for more accurately determining the energy requirements of these patients.

**Objective:**

The primary objective of this study was to compare the REE, increased by 20% (to account for diet-induced thermogenesis and daily energy expenditure), as measured by IC in patients at different stages of CD, with the total energy expenditure (TEE) calculated using the Simple Weight-Based Equation (30 kcal/kg and 35 kcal/kg).

**Methods:**

Sessions for measuring REE were conducted using IC, along with the collection of clinical, biochemical, and anthropometric data. The clinical activity of CD was classified using the Harvey-Bradshaw Index (HBI), while endoscopic classification was assessed through the Simple Endoscopic Score in CD (SES-CD).

**Results:**

A total of 60 adult patients diagnosed with CD in different disease phases were randomly evaluated, with 56.7% being male and 43.3% female, and a mean age of 39 years. The majority (76.7%) were of caucasian ethnicity, and 48.3% had completed high school. According to body mass index (BMI), 21.7% were classified as undernourished, 48.3% as eutrophic, 16.7% as overweight, and 13.3% as obese. Regarding disease activity classification based on the HBI, 50% were in the active phase and 50% in remission. Endoscopic classification revealed that 60% had findings indicative of active disease. The patients evaluated were diagnosed with Crohn’s disease (CD) at an average age of 28.2 years, with the majority presen­ting ileocolonic involvement (61.8%) and a stenosing behavior (45.5%). Regarding biochemical serum analysis, the average values found were 12.5 g/dL for hemoglobin, 38.7% for hematocrit, and 15.7 mg/L for C-reactive protein. 58.3% of patients did not have fistulas. No agreement was found between the energy expenditure results measured by the weight-based formulas (30 kcal/kg and 35 kcal/kg) and IC (ICC <0.4), with the values obtained by simple weight-based equations being higher than those from IC. The result obtained using 30 kcal/kg showed slightly greater concordance with IC, but still with low agreement. In isolation, energy expenditure in male patients was statistically higher than in female patients. There was a statistically significant direct correlation between energy expenditure and hemoglobin levels, as well as statistically significant indirect correlations with age and age at diagnosis. The difference in energy expenditure between the methods was indirectly correlated with age, BMI, and age at diagnosis. No statistically significant correlations were found between energy expenditure and the behavior, location, or activity of Crohn’s disease.

**Conclusion:**

This study suggests that the use of IC would be more beneficial for younger patients, with lower BMI, or those diagnosed at a younger age, given the greater discrepancy between the methods evaluated in these cases. For other patients, the 30 kcal/kg weight-based formula remains a practical and more accessible option. However, it is undeniable and increasingly recognized that IC provides a more accurate assessment of energy requirements in different clinical conditions, and when used appropriately, it can enhance nutritional support and care.

## HIGHLIGHTS

• Analysis of the correlation between resting energy expenditure and clinical characteristics of patients with Crohn’s disease, comparing different energy estimation methods

• Individuals diagnosed with Crohn’s disease, aged 18 years or over, of both sexes, were included in this study.

• Simple Weight-based Equations overestimated total energy expenditure compared to indirect calorimetry.

• Indirect calorimetry is recommended for certain patients, including younger individuals, those with a lower BMI, or those diagnosed early, due to greater discrepancies between methods and the risk of inadequate caloric intake with weight-based equations.

## RESUMO

**Contexto:**

Os pacientes diagnosticados com doença de Crohn (DC) apresentam alto risco de comprometimento do estado nutricional em decorrência dos sintomas e da intensa resposta inflamatória da doença e a utilização da calorimetria indireta (CI) para a aferição do gasto energético de repouso (GER) se mostra uma boa alternativa para melhor quantificar as necessidades energéticas desses pacientes.

**Objetivo:**

O principal objetivo desse estudo foi comparar o GER, acrescido de 20% (termogênese da dieta e dispêndio energético diário), mensurado pela CI, dos pacientes nas diferentes fases da DC, com o Gasto Energético Total (GET) calculado pela Fórmula de Bolso (FB) (30 kcal/kg e 35 kcal/kg).

**Métodos:**

Foram realizadas sessões de aferição do GER, utilizando a CI, além da coleta de dados clínicos, bioquímicos e medidas antropométricas. Para classificação de atividade clínica da DC foi utilizado o Índice de Harvey-*Bradshaw* (HBI), e a classificação endoscópica foi mensurada através do *Simple Endoscopic Score in CD* (SES-CD).

**Resultados:**

Foram avaliados ao acaso, 60 pacientes adultos diagnosticados com DC nas diferentes fases da doença, 56,7% do sexo masculino e 43,3% do sexo feminino, com idade média de 39 anos, sendo a maioria (76,7%) brancos e possuíam o ensino médio completo (48,3%). De acordo com o índice de massa corporal (IMC), 21,7% foram classificados em desnutrição, 48,3% em eutrofia, 16,7% em sobrepeso e 13,3% em obesidade. Quanto a classificação de atividade clínica da doença segundo o HBI, 50% estavam em atividade e 50% em remissão. Para classificação endoscópica, 60% possuíam achados de atividade de doença. Os pacientes avaliados receberam o diagnóstico de DC em média aos 28,2 anos, em sua maioria com acometimento ileocolônico (61,8%) e comportamento estenosante (45,5%). Em relação aos resultados de análises bioquímicas séricas, foi encontrado um valor médio de 12,5 g/dL para hemoglobina, 38,7% para hematócrito e 15,7 mg/L para proteína C reativa. 58,3% dos pacientes não possuíam fístulas. Não foi encontrada concordância entre os resultados do gasto energético aferido pela FB a 30 kcal/kg e 35 kcal/kg com a CI (CCI < 0,4), sendo os valores encontrados pela FB maiores que os da CI. O resultado da FB a 30 kcal/kg foi um pouco mais concordante com a CI, mas ainda com concordância baixa. Isoladamente, o gasto energético nos homens foi estatisticamente maior que nas mulheres. Houve correlação direta estatisticamente significativa do gasto energético com a hemoglobina e correlações indiretas estatisticamente significativas com a idade e a idade de diagnóstico. A diferença no gasto energético entre os métodos foi indiretamente correlacionada com a idade, IMC e idade no diagnóstico. Não foram encontradas correlações estatisticamente significativas entre o gasto energético e comportamento, localização e atividade da DC.

**Conclusão:**

Este estudo sugere que o uso da CI seria mais bem aproveitado em pacientes jovens, com menor IMC ou diagnosticados mais novos, considerando a maior diferença entre os métodos avaliados para estes casos. Para os demais pacientes a FB a 30 kcal/kg continua sendo uma opção prática e mais acessível. Entretanto, é inegável e cada vez mais reconhecido que a CI fornece uma avaliação mais precisa da demanda energética nas diferentes condições clínicas e, quando usada adequadamente, pode melhorar o suporte e cuidado nutricional.

## INTRODUCTION

Crohn’s disease (CD) is an inflammatory bowel disease (IBD) characterized by transmural, segmental, and asymmetric lesions, which can affect any part of the digestive tract. However, it is most commonly observed in the terminal ileum and colon^1^. While the disease is more frequently diagnosed in young individuals under the age of 30, studies show a significant increase in its incidence among older populations both in Brazil and globally^2^ ^,^ ^3^. Although its etiology is not fully understood, several factors may be associated with the development of CD, including genetic susceptibility, immune response, environmental factors, and an imbalance in the gut microbiota^1^.

CD is characterized by periods of activity and remission, with clinical manifestations varying depending on the location and extent of the lesions. Symptoms can include chronic diarrhea, recurrent abdominal pain, fever, anorexia, malabsorption, and weight loss. Additionally, between 21% and 47% of patients may present with extra-intestinal systemic manifestations (EIMs), which can be of dermatological, ocular, thromboembolic, and bone origins, among others^4^.

The diagnosis of CD is challenging, as many patients are asymptomatic, and when symptoms are present, they can vary significantly between individuals^5^. To confirm the diagnosis, endoscopic and radiological evaluations are essential, along with complementary laboratory tests, such as hemoglobin, hematocrit, C-reactive protein, and fecal calprotectin, to assess disease severity^6^. 

Currently, the most common clinical methods for evaluating disease activity include the Crohn’s Disease Activity Index (CDAI) and the Harvey-Bradshaw Index (HBI), with the latter being easier to apply^7^ ^,^ ^8^.

To measure the endoscopic activity of the disease, scores such as the Crohn’s Disease Endoscopic Index of Severity (CDEIS)^9^ or the Simple Endoscopic Score for Crohn’s disease (SES-CD)^10^ can be used. Both were developed with the aim of evaluating prognosis and analyzing treatment effectiveness^11^.

In the pursuit of a more accurate classification of patients, the Montreal classification scale^12^ was developed, which standardizes the main phenotypic aspects of the disease, taking into account the age at diagnosis in years, the anatomical location of the disease, and its behavior.

The complex pathophysiology of CD induces structural and functional changes in the intestine, resulting in nutrient malabsorption, weight loss, and increasing catabolism within this population. Additionally, the intense inflammatory response of the disease, especially during active phases, leads to an increase in resting energy expenditure (REE), making it difficult to meet energy and protein requirements. As a result, malnutrition is a common outcome in patients with CD, and several studies associate its presence with worse prognosis, increased complications and mortality, as well as a significant reduction in quality of life^13^. 

Malnutrition is a frequent complication in IBDs, but its prevalence is exceptionally high in CD, possibly due to the disease’s ability to affect any part of the gastrointestinal tract, with a focus on the small intestine, which is responsible for absorbing a large portion of nutrients^13^. In this sense, nutritional assessment plays a crucial role in the healthcare of these patients, as they are at high risk of nutritional impairment, with energy-protein malnutrition being diagnosed frequently^14^.

As an essential part of nutritional assessment and management, it is necessary to estimate the patient’s energy and protein needs with the highest possible level of accuracy in order to establish appropriate nutritional strategies. For this purpose, there are several methods available, including indirect calorimetry (IC) and the simple weight-based equation^15^.

IC is an effective, non-invasive method and is considered the gold standard for measuring REE due to its accurate results. The estimation of REE is made through the measurement of oxygen (VO2) and carbon dioxide production (VCO2) in an individual, calculated using Weir’s equation [(VO2L/min x 3.94) + (VCO2L/min x 1.11) x 1440]. However, due to the high cost of the equipment, maintenance, and the need for trained team to perform the test, few healthcare services in Brazil routinely offer its availability^16^.

As an alternative to this difficulty, energy requirements be estimated through the simple weight-based equation, which involves multiplying a fixed value by the patient’s weight. This method is popular because it is practical and widely accepted in clinical practice. However, despite its simplicity, the simple weight-based equation has lower accuracy, which can lead to insufficient or excessive caloric intake in some cases. The guidelines of the European Society for Clinical Nutrition and Metabolism (ESPEN) recommend a daily intake range of 30 to 35 kcal/kg of body weight for adults diagnosed with IBD. When conducting an initial nutritional assessment in a patient, it is generally considered appropriate to calculate 30 kcal/kg of the appropriate reference weight, with subsequent adjustments to the caloric target made based on the clinical response^14^. 

In this regard, given the nutritional risk of patients with CD and aiming to avoid inadequate caloric intake that could impact weight loss and worsen prognosis, it is imperative to seek new evidence that provides a more solid professional basis for choosing the method of measuring energy expenditure. This would establish its correlation with disease activity, enabling the implementation of specific interventions that result in improvements in quality of life and disease outcomes.

## OBJECTIVE

### General

To compare the REE, increased by 20% (diet-induced thermogenesis and daily energy expenditure), measured by IC, in patients with active and remission phases of CD, with the total energy expenditure (TEE) calculated using the simple weight-based equations.

### Specific

- To compare the TEE of patients in active or remission phases of CD;
- To assess the correlation between the analyzed characteristics and the TEE;
- To evaluate the most accurate method for measuring energy expenditure according to the analyzed characteristics.

## METHODS

A non-randomized clinical trial was conducted, involving adult patients diagnosed with CD in either the active or remission phase. The study was carried out at the IBD clinic of the Central Institute of the Hospital das Clínicas of the Faculty of Medicine, University of São Paulo (CI-HCFMUSP) from August 2022 to October 2023.

The recruitment of participants was conducted by the principal investigator, who approached patients randomly during their visits to the clinic, inviting them to participate in the research. The objectives, benefits, risks, and procedures involved in data collection were explained to the participants, and the informed consent form was signed.

Individuals aged 18 years and older, regardless of gender, who have been diagnosed with CD and are currently being monitored at the clinic, were included in the study. Patients were excluded if they met any of the following criteria: pregnant women, individuals under 18 years old, those with a history of chronic liver disease, diabetes mellitus, thyroid dysfunction, HIV, tuberculosis, cancer, or other acute or chronic illnesses. Additionally, patients who had undergone surgery within the past three months or who did not have a fully established diagnosis of CD were also excluded from the study.

After agreeing to participate in the study, a day and time were scheduled for the participants to attend the clinic, where they underwent the IC test. Prior to the test, the individuals received specific instructions, including the need for a four-hour fast, covering food and beverages (including water), abstaining from alcohol and caffeine consumption (such as coffee, tea, soda, chocolate, and energy drinks) for 48 hours, and the recommendation to avoid physical activity 24 hours before the test.

Initially, a questionnaire was applied to obtain the patient profile, and measurements of weight and height were taken to calculate the body mass index (BMI) and assess the nutritional status. Patients were classified using the Montreal Classification scale, according to the age at diagnosis, location, and behavior of the disease. To classify the clinical activity of CD, the HBI was used, considering disease activity for patients who scored 5 or higher.

The endoscopic classification was measured using the Simple Endoscopic Score in CD (SES-CD) based on a recent colonoscopy performed within one year prior to the date of the IC test. The laboratory activity of the disease was assessed through the results of serum biochemical analyses of hemoglobin, hematocrit, and the biomarker C-reactive protein, which were obtained from the electronic medical records.

For the evaluation of REE, the IC (Q-NRG+, COSMED) was chosen as it is recognized as a reference method for determining the number of kilocalories necessary to maintain the proper functioning of vital organs at rest (functioning of the cardiovascular and respiratory systems, and thermoregulatory mechanisms responsible for regulating body temperature). The IC test was performed with the patient in a supine position, under standard resting conditions, immobility, thermoneutrality (22°C-24°C), and relaxation. The IC session was completed after 15 minutes from the start of the test, and the REE was calculated using the simplified Weir equation [(VO2L/min x 3.94) + (VCO2L/min x 1.11) x 1440], thus not considering urinary nitrogen.

For the purpose of comparison, when measuring the REE using IC, an additional 20% was added, representing the thermic effect of food and the daily energy expenditure. His inclusion was made to facilitate the comparison with the result of the calculation of energy needs using the simple weight-based equations (kcal/kg). This approach is adopted because the TEE includes not only REE but also takes into account the thermic effect of food (diet-induced thermogenesis) and the energy expenditure related to performing external mechanical work (daily energy expenditure)^17^.

For statistical analysis, the data were analyzed using the IBM-SPSS software for Windows version 22.0 and tabulated using Microsoft Excel 2013. Qualitative characteristics evaluated in all patients were described using absolute and relative frequencies, while quantitative characteristics were described using summary measures (mean, standard deviation, median, and quartiles). Intraclass correlation coefficients with their respective 95% confidence intervals and the repeatability measure were calculated to assess the agreement between the energy expenditure evaluation methods and estimate the difference between the methods, respectively.

The values and difference in energy expenditure between methods were described according to qualitative characteristics using summary measures and compared using t-student test for only 2 categories, or analysis of variance (ANOVA) for more than 2 categories. Pearson correlations of energy expenditure and the difference in energy expenditure between the methods with the quantitative characteristics were calculated to assess the existence of a correlation between the characteristics and the outcomes. Only for the C-reactive protein was Spearman’s correlation used due to the non-normal distribution of this variable.

Multiple linear regression models for energy expenditure and the difference in energy expenditure between the methods were created using the characteristics that were statistically significant in the unadjusted analyses to jointly verify which characteristics influence the outcomes. The coefficient of determination (R²) of the models was presented to assess how much of the outcomes were explained by the adjusted characteristics. The tests were performed with a significance level of 5%.

## RESULTS AND DISCUSSION

Sixty adult patients diagnosed with CD in different stages of the disease were evaluated at the IBD clinic of the Central Institute of the *Hospital das Clínicas* of the Faculty of Medicine, University of São Paulo (CI-HCFMUSP).

Among the participants, 56.7% were male and 43.3% were female, with an average age of 39 years. The majority of the patients (76.7%) were of caucasian ethnicity and had completed high school (48.3%). Regarding nutritional status, according to the BMI, 21.7% were classified as malnourished, 48.3% as eutrophic, 16.7% as overweight, and 13.3% as obese. According to the CDAI, 50% of patients were in active disease, while 50% were in remission. In the endoscopic classification, 60% had findings indicative of disease activity. The average age at diagnosis of CD was 28 years, with the majority of patients having ileocolonic involvement (61.8%) and a stenosing disease behavior (45.5%). Most of the patients (58.3%) did not have fistulas. Regarding the results of serum biochemical analyses, the mean values were 12.5 g/dL for hemoglobin, 38.7% for hematocrit, and 15.7 mg/L for C-reactive protein (Table 1).

**TABLE 1:** Description of the characteristics of all patients.

Variable	Description (N=60)
Age (years)	
mean ± SD	38.8±15.6
median (p25; p75)	37.5 (25; 48)
Gender, n (%)	
Male	34 (56.7)
Femeale	26 (43.3)
Ethnicity, n (%)	
Caucasian	46 (76.7)
Black	14 (23.3)
Education level, n (%)	
Middle School	16 (26.7)
High School	29 (48.3)
Higher Education	15 (25)
BMI (kg/m²)	
mean ± SD	23.1±4.9
median (p25; p75)	21.8 (19.4; 26.2)
BMI Classification, n (%)	
Undernutrition	13 (21.7)
Eutrophic	29 (48.3)
Overweight	10 (16.7)
Obesity	8 (13.3)
Clinical classification (HBI), n (%)	
Disease activity	30 (50)
Disease remission	30 (50)
Colonoscopy result, n (%)	
Disease activity	36 (60)
Disease remission	24 (40)
Disease location, n (%)&	
Ileal	12 (21.8)
Ileocololonic	34 (61.8)
Colonic	9 (16.4)
Disease behavior, n (%)&	
Inflammatory	18 (32.7)
Fistulizing	12 (21.8)
Stricturing	25 (45.5)
Age at diagnosis (ears)&	
mean ± SD	28.2±13.6
median (p25; p75)	25 (18; 35)
Hemoglobin (g/dL)*	
mean ± SD	12.5±2.1
median (p25; p75)	12.7 (11.3; 13.8)
Hematocrit (%)&	
mean ± SD	38.7±5.5
median (p25; p75)	39.7 (36.1; 41.8)
C-reactive protein (mg/L)&	
mean ± SD	15.7±21.2
median (p25; p75)	7.9 (2.4; 19.4)
Fistula, n (%)	
No	35 (58.3)
Yes	25 (41.7)
Diference (REE+20% - 30 kcal/kg)	
mean ± SD	-162.3±420.2
median (p25; p75)	-115.2 (-372.3; 166)

*some patients do not have the information.

According to the latest guideline from the European Society for Clinical Nutrition and Metabolism (ESPEN), from 2023, the energy requirement for patients with CD is considered similar to that of the general population. The use of IC is recommended when there is clinical suspicion of a higher individual demand, with a caloric intake recommendation between 30 and 35 kcal/kg of body weight^14^. However, the results of this study present discrepancies in relation to this recommendation.

Analyzing Table 2, it is observed that neither the simple weight-based equations with 30 kcal/kg nor with 35 kcal/kg were in agreement with the IC results (intraclass correlation coefficient [ICC] <0.4). The result for the simple weight-based equations with 30 kcal/kg was slightly more concordant with the CI, but still with low agreement.

**TABLE 2:** Description of energy expenditure according to each assessment method and result of the concordance between the calorimetry method and the pocket formula.

Variable	Description	ICC	CI (95%)		Repeatability
	(N=60)		Inferior	Superior	
REE + 20%					
mean ± SD	1800.9±418.5				
median (p25; p75)	1837.2 (1552.8; 2055.3)				
30 kcal/kg		0.398	0.162	0.590	297.2
mean ± SD	1963.3±365.3				
median (p25; p75)	1963.5 (1677; 2235)				
35 kcal/kg		0.260	-0.073	0.534	318.4
mean ± SD	2290.5±426.2				
median (p25; p75)	2290.8 (1956.5; 2607.5)				

ICC: intraclass correlation coefficient; CI: confidence interval.

In this sense, the results indicate that the Simple weight-based equations may be overestimating energy expenditure compared to measurement by IC. Patients with CD are prone to nutritional impairment, and the changes in intestinal absorption alterations observed in these cases are not easily quantifiable through energy expenditure measurement.

Given the frequent need to restore nutritional status, especially in undernourished individuals, a caloric intake ranging from 30 to 35 kcal/kg of body weight proves to be appropriate for this purpose. Additionally, it is important to emphasize the need for continuous monitoring of energy balance and physical activity levels through outpatient follow-up to assess the patient’s response to nutritional treatment, allowing the nutritionist to make necessary adjustments as the clinical condition evolves.

Insufficient energy intake can contribute to malnutrition and consequent unfavorable clinical outcomes. On the other hand, to prevent excessive nutrient intake, it is crucial to be aware of potential glycemic alterations, liver dysfunctions, dyslipidemia, and electrolyte disturbances, particularly in hospital settings^18^. According to the American Society for Parenteral and Enteral Nutrition (ASPEN), studies have shown that the simple weight-based equations with a caloric intake of 25 kcal/kg approximated the energy expenditure values obtained by IC and did not prove inferior to the gold-standard method for maintaining body weight^19^.

In the literature, there are few studies exploring the measurement of energy expenditure n patients with CD through CI compared to other predictive methods, with existing studies using equations different from the simple weight-based equations^20^ ^-^ ^23^. Among these studies, some data indicate an inverse correlation between TEE and and the CDAI, meaning that as the severity of inflammation increases, TEE tends to decrease^24^. This correlation may be explained by changes in body composition caused by inflammation, use of corticosteroid medications, and reduction in daily physical activity levels^20^.

Table 3 presents the description of energy expenditure according to qualitative characteristics, the results of comparative tests, and the correlation of energy expenditure with quantitative characteristics. The data show that, on isolation, energy expenditure in men was on average statistically higher than in women, a finding that is consistent with what is widely documented in the literature. It is known that TEE is primarily influenced by factors such as physical activity level, food intake, weight, and body composition^15^. Given that muscle tissue is metabolically active, it plays a significant role in this calculation^15^. Therefore, it is suggestive that male individuals, due to their higher muscle mass content compared to women, naturally exhibit a higher TEE.

**TABLE 3:** Description of energy expenditure according to qualitative characteristics and results of comparative tests, as well as the correlation of energy expenditure with quantitative characteristics.

Variable	REE + 20%	*P*
Age (years)	-0.294	0.023
Gender		0.023**
Male	1907.6±442.4	
Female	1661.5±345.5	
Ethnicity		0.158**
Caucasian	1758.7±380.9	
Black	1939.8±515.6	
Education level		0.186£
Middle School	1801.7±514.8	
High School	1884.1±389.9	
Higher Education	1639.4±328.7	
BMI (kg/m²)	0.185	0.158
Clinical classification (HBI)		0.731**
Disease activity	1782.2±399	
Disease remission	1819.7±443.2	
Colonoscopy result		0.380**
Disease activity	1761.8±396.2	
Disease remission	1859.6±452.2	
Disease location		0.506£
Ileal	1865.9±322	
Ileocololonic	1779.6±410.7	
Colonic	1944.3±433.4	
Disease behavior		0.373£
Inflammatory	1922.9±315.8	
Fistulizing	1720.2±480.2	
Stricturing	1805.7±402.2	
Age at diagnosis (years)	-0.289	**0.026**
Hemoglobin (g/dL)	0.307	**0.020**
Hematocrit (%)	0.242	0.070
CRP (mg/L)	0.053	0.699
Fístula		0.134**
No	1869.5±398.7	
Yes	1704.9±434.7	

Data expressed as mean ± SD or correlation; Pearson’s correlation; &:Spearman’s correlation; **Unpaired t-student; £ ANOVA.

A statistically significant direct correlation was also observed between energy expenditure and serum hemoglobin levels. In other words, as hemoglobin levels increase, the patient’s energy expenditure also increases. However, it is important to emphasize that this correlation still requires a deeper understanding, as it may be influenced by various factors that need to be elucidated.

Additionally, statistically significant indirect correlations were identified with age and age at diagnosis. Thus, younger patients or those diagnosed with CD at an earlier age had higher energy expenditure. This observation can be explained by the fact that, as we age, the TEE tends to decrease due to changes in body composition related to an increase in fat mass and a reduction in lean mass, which directly impacts energy expenditure^17^.

No statistically significant correlations were found between TEE and disease behavior, location, and activity.

Evaluating the description of the difference in energy expenditure between methods and its correlation with the characteristics (Table 4), it was found that, in isolation, the difference in energy expenditure between methods was significantly inversely correlated with age, BMI, and age at diagnosis, meaning that the younger the age, the lower the BMI, and the earlier the diagnosis, the greater the difference found between the methods.

**TABLE 4:** Description of the difference in energy expenditure between methods according to qualitative characteristics, results of comparative tests, and correlation of energy expenditure with quantitative characteristics.

Variable	Diference (REE+20%-30 kcal/kg)	*P*
Age (years)	-0.530	**<0.001**
Gender		0.360**
Male	-118.5±422.8	
Female	-219.6±418.1	
Ethnicity		0.592**
Caucasian	-146.1±416.9	
Black	-215.6±442.6	
Education level		0.218£
Middle School	-262.7±507	
High School	-64.3±393.4	
Higher Education	-244.8±347.3	
BMI (kg/m²)	-0.514	**<0.001**
Clinical classification (HBI)		0.294**
Disease activity	-104.9±466.5	
Disease remission	-219.8±367.3	
Colonoscopy result		0.359**
Disease activity	-121.3±419.2	
Disease remission	-223.9±423.1	
Disease location		0.085£
Ileal	-22.4±370.4	
Ileocololonic	-223.3±440.5	
Colonic	76.1±200.5	
Disease behavior		0.793£
Inflammatory	-179.1±362.6	
Fistulizing	-75.8±553	
Stricturing	-121.7±374.9	
Age at diagnosis (years)	-0.521	**<0.001**
Hemoglobin (g/dL)	0.039	0.771
Hematocrit (%)	0.006	0.966
CRP (mg/L)	0.216	0.111
Fístula		0.701**
No	-144.5±375.3	
Yes	-187.2±483.3	

Data expressed as mean ± SD or correlation; Pearson’s correlation; &: Spearman’s correlation; **Unpaired t-student; £ ANOVA.

In this context, it is understood that the discrepancy between methods is more pronounced in younger patients, those with a lower BMI, or those who were diagnosed with CD at an earlier age. Therefore, it is suggested that the use of IC may be more beneficial in this population, as using the Simple weight-based equations could lead to significant variations in in results between the methods, resulting in an inadequate energy intake.

Table 5 shows that, regardless of other patient characteristics, the energy expenditure was, on average, 7.8 points lower for each additional year in the age of diagnosis, and increased by 58 points for each 1 g/dL increase in hemoglobin. On the other hand, the difference in energy expenditure between the methods was, on average, 11.3 points lower for each additional year in the age of diagnosis, and decreased by 30.1 points for each 1 kg/m² increase in the patient’s BMI. The variability in energy expenditure was explained by only 17.1%, considering the patient’s age at diagnosis and serum hemoglobin. Meanwhile, the variability in the difference in energy expenditure between methods was explained by 36.9%, using the age at diagnosis and BMI of the patients.

**TABLE 5:** Results of the models to explain energy expenditure and the difference in energy expenditure between methods based on patient characteristics.

Outcome	Variable	Coefficient	Standard Error	*t Value*	*P*	R²
REE + 20%	Constant	1281.6	348.5	3.68	0.001	0.171
	Age at diagnosis (years)	-7.8	3.7	-2.12	0.039	
	Hemoglobin (g/dL)	58.0	25.6	2.26	0.028	
Diference (REE+20%-30 kcal/kg)	Constant	852.5	216.6	3.94	<0.001	0.369
	Age at diagnosis (years)	-11.3	3.7	-3.05	0.003	
	BMI (kg/m²)	-30.1	10.3	-2.93	0.005	

Multiple Linear Regression.

The test discussed is challenging to perform and is not widely available in healthcare services across the country. The study suggests that its use would be better optimized for younger patients, those with a lower BMI, or individuals diagnosed at earlier ages. This is because there is a larger discrepancy between the methods used in these cases, along with the potential risk of inadequate caloric intake when applying the simple weight-based equations.

For other patients, however, the differences observed are smaller. This indicates that using the Simple Weight-based Equations at 30 kcal/kg remains a practical and more accessible option. Nonetheless, in situations where there is suspicion of a specific energy demand, IC remains the most suitable method for assessing REE.

## CONCLUSION

No agreement was found between energy expenditure results from simple weight-based Equations (30 and 35 kcal/kg) and IC, with the equations yielding higher values. The 30 kcal/kg equation showed slightly better concordance with IC but remained low. Men had statistically higher energy expenditure than women.

Factors like age, BMI, and age at diagnosis influenced TEE differences, with greater discrepancies in younger, lower BMI, and earlier-diagnosed patients. Energy expenditure correlated directly with serum hemoglobin levels.

Due to IC’s limited feasibility, its use is recommended for patients with higher discrepancies, while the 30 kcal/kg equation remains a practical alternative. However, IC remains the most accurate method when specific energy needs are suspected. Selecting the appropriate assessment method is crucial to ensuring adequate caloric intake and preventing nutritional risks.
